# Long-term results of the metaphyseal-loading anterolaterally flared anatomic femoral stem for total hip arthroplasty

**DOI:** 10.1007/s00264-025-06624-y

**Published:** 2025-09-06

**Authors:** Makoto Kitade, Tetsuro Tani, Akihiko Matsumine

**Affiliations:** 1 University of Fukui, Fukui, Japan; 2 Osaka University, Osaka, Japan

**Keywords:** Osteoarthritis, Hip, Survival rate, Developmental hip dysplasia

## Abstract

**Background:**

Patients with secondary hip osteoarthritis due to developmental dysplasia of the hip (DDH) often have abnormal femoral morphology, making stem design critical for long-term outcomes. The FMS-anatomic stem previously demonstrated favourable mid-term results. Its successor, the Anatomic Fit stem, was developed with a reduced hydroxyapatite-coated area to enhance proximal load transfer and a narrower lateral flare to facilitate insertion. This study aimed to analyze the long-term clinical and radiographic outcomes of these stems.

**Methods:**

The one-hundred seventy-eight hips underwent total hip arthroplasty using either the FMS-anatomic or Anatomic Fit stem. After applying exclusion criteria, 119 hips (36 FMS-anatomic and 83 Anatomic Fit) were analyzed. Clinical and radiographic outcomes were assessed, and stem survival was evaluated using Kaplan–Meier analysis with stem revision as the endpoint.

**Results:**

The 15- and 20-year survival rates were 97.2% and 94.4% for the FMS-anatomic stem, and 98.8% for the Anatomic Fit stem at both time points, with no significant difference. Spot welds were observed in nearly all cases, but their distribution differed significantly: zones 2 and 6 in the FMS-anatomic group and zones 1 and 7 in the Anatomic Fit group (*P* < 0.05). Stress shielding of Grade 3 or higher occurred in 16.5% of FMS-anatomic stems and 33.7% of Anatomic Fit stems (*P* < 0.05).

**Conclusions:**

Both stems showed excellent long-term survival and proximal fixation. However, the Anatomic Fit stem did not reduce stress shielding, despite its modified design intended to improve load transfer.

## Introduction

Total hip arthroplasty (THA) is a common procedure for end-stage hip osteoarthritis (HOA) [1], providing significant pain relief and functional improvement. Developmental dysplasia of the hip (DDH) is prevalent in over 81% of Japanese patients with secondary HOA [2], often presenting with abnormal femoral bone morphologies such as varus, valgus, and anteversion [3–5]. In cementless stems, initial fixation relies on cortical bone contact, and porous surfaces facilitate bone ingrowth for long-term stability [6].

Previously, we analyzed the proximal femoral shape in Japanese patients with secondary HOA due to DDH [7–9], leading to the development of the FMS-anatomic stem (KYOCERA Medical, Osaka, Japan), a proximally fitting, anterolaterally flared stem with two different medial curves, a lateral shoulder, and a proximal hydroxyapatite coating over an arc-deposited titanium plasma spray. This stem has been in use since 1998 [7, 8, 10, 11], demonstrating favourable mid-term outcomes [12].

Finite element analysis indicated load transfer occurs at spot welds between cortical bone-stem contact points [13]. Consequently, the Anatomic Fit stem was developed, featuring a 35% reduction in proximal hydroxyapatite coating for enhanced proximal fixation, a 2 mm reduction in lateral flare for easier insertion and reduced fracture risk, and a polished distal end to prevent distal fixation. This stem was used from 2003. This study aims to evaluate the survival rates of the FMS-anatomic and Anatomic Fit stems and assess whether the design modifications yield improved clinical outcomes.

## Methods

### Study design

**Fig. 1:**
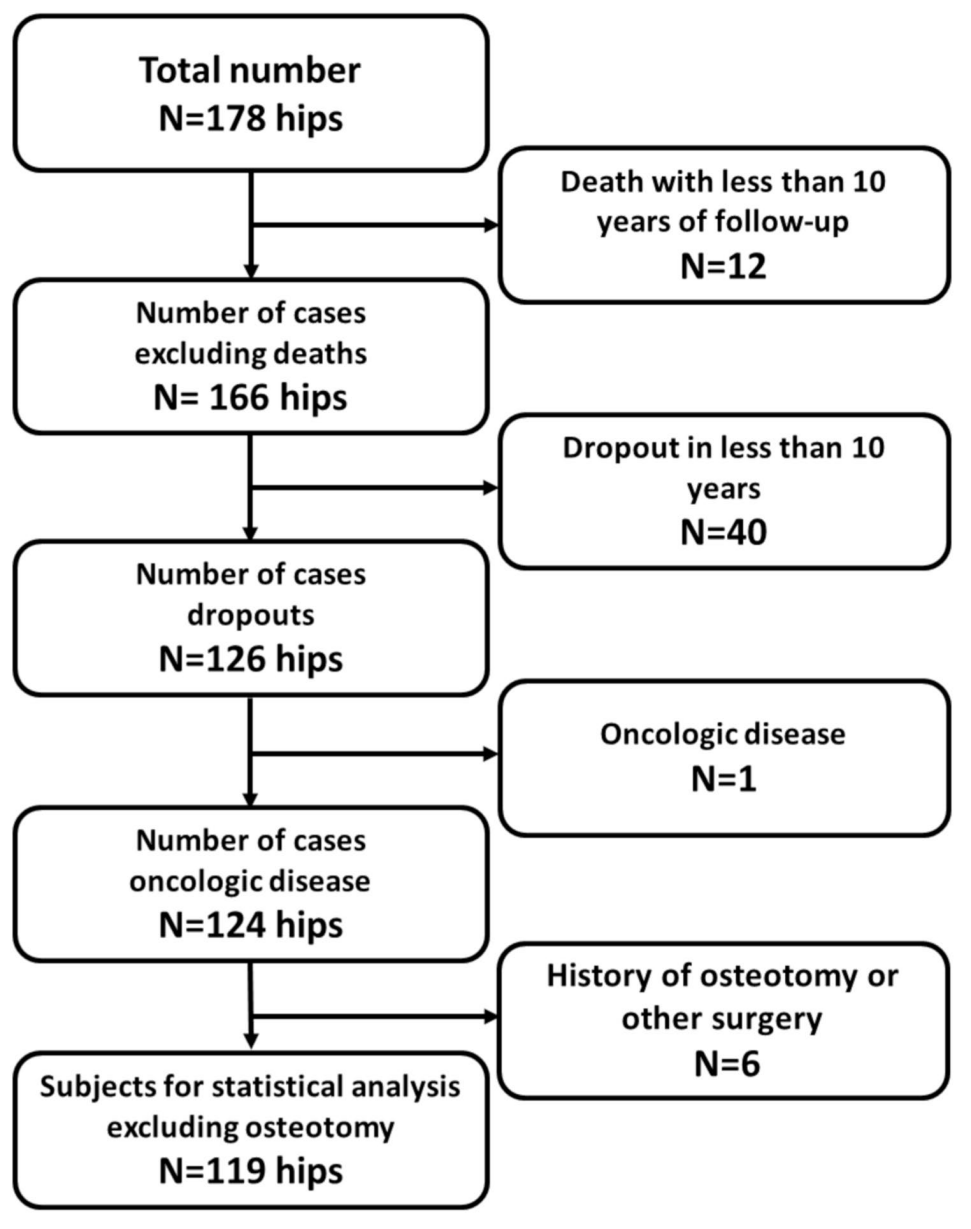
Excluding map. THA performed using the two applicable stem types (FMS anatomic stem and anatomic fit stem) in our hospital between 1998 to 2014

The ethics committee of our university approved this retrospective case series. A total of 178 hips that underwent primary THA with either the FMS-anatomic or Anatomic Fit stems were initially selected between April 1998 and March 2014. We excluded 12 hips due to patient death and 40 hips due to lost follow-up within ten years, leaving 126 hips (follow-up rate, 70.8%). Six hips with prior osteotomy of the femur or pelvis and one hip with a tumor affecting the femur were further excluded. Thus, the final cohort comprised 119 hips from 99 patients (81 women, 18 men; mean age: 59.1 years [range, 30 to 84]) (Fig. 1). The underlying diagnoses were secondary HOA due to DDH in 97 patients, osteonecrosis of the femoral head in eight patients, and primary HOA in six patients. The mean follow-up period was 15.3 years (10–25.6 years). The FMS-anatomic stem was used in 36 hips between 1998 and 2003, while the Anatomic Fit stem was used in 83 hips between 2004 and 2014 (Table 1). The preoperative Dorr classification for the FMS-anatomic stem was Type A in 24 hips (66.7%) and Type B in 12 hips (33.3%), with no Type C hips. For the Anatomical Fit stem, Type A was observed in 43 hips (51.8%), Type B in 37 hips (44.6%), and Type C in three hips (3.6%) (Table 1). There was no significant difference between the two stems in the Dorr classification, but the Anatomic Fit tended to be used for Dorr B and C. During surgery, straight reamers with dull tips were used before broaching to enhance the fit and fill of the prosthesis, as opposed to a “broach-only” technique [14]. The AMS HA cup (Kyocera Medical, Warsaw, IN) was used in 118 patients (99.2%). For inserts, polyethylene and AMS liners (Kyocera Medical, Warsaw, IN) were used in 106 cases (89.1%), ABS liners (Kyocera Medical, Warsaw, IN) in 11 cases (9.2%), and an Omnifit liner (Stryker Howmedica Osteonics, Mahwah, NJ) in one case (0.8%). Metal and PHS heads (Kyocera Medical, Warsaw, IN) were used in all cases. All surgeries were performed by three surgeons using a posterolateral approach under general anaesthesia.

**Table 1:** Demographics of patient groups

Variable	Total	FMS-anatomic stem	Anatomic Fit stem	*P*-value (FMS-anatomic stem vs. Anatomic Fit stem)
Number of hips	119 hips	36 hips	83 hips	
Age(years)	59.0 ± 9.45	56.8 ± 10.16	60.0 ± 9.02	0.09
Sex(%)				
Women	84.9(101/119)	75.0(27/36)	89.1(74/83)	< 0.05
Men	15.1(18/119)	25.0(9/36)	10.8(9/83)	
BMI	23.9 ± 3.71	23.9 ± 4.01	23.9 ± 3.60	0.96
Disease(%)				0.70
DDH	77.3(92/119)	76.9(30/36)	84.3(70/83)	
ONFH	6.7(8/119)	8.3(3/36)	6.0(5/83)	
Primary	5.0(6/119)	2.8(1/36)	6.0(5/83)	
Others	4.2(5/119)	5.6(2/36)	3.6(3/83)	
Dorr type(%)				0.21
A	56.3(67/119)	66.7(24/36)	51.8(43/83)	
B	41.1(49/119)	33.3(12/36)	44.6(37/83 )	
C	2.5(3/119)	0(0/36)	3.6(3/83)	

Values are expressed as mean ± standard deviation or number (%)

BMI, body mass index; DDH, developmental dysplasia of the hip; ONFH, osteonecrosis of the femoral head

*t* test or Fisher’s exact test was used

### Femoral stem design

**Fig. 2:**
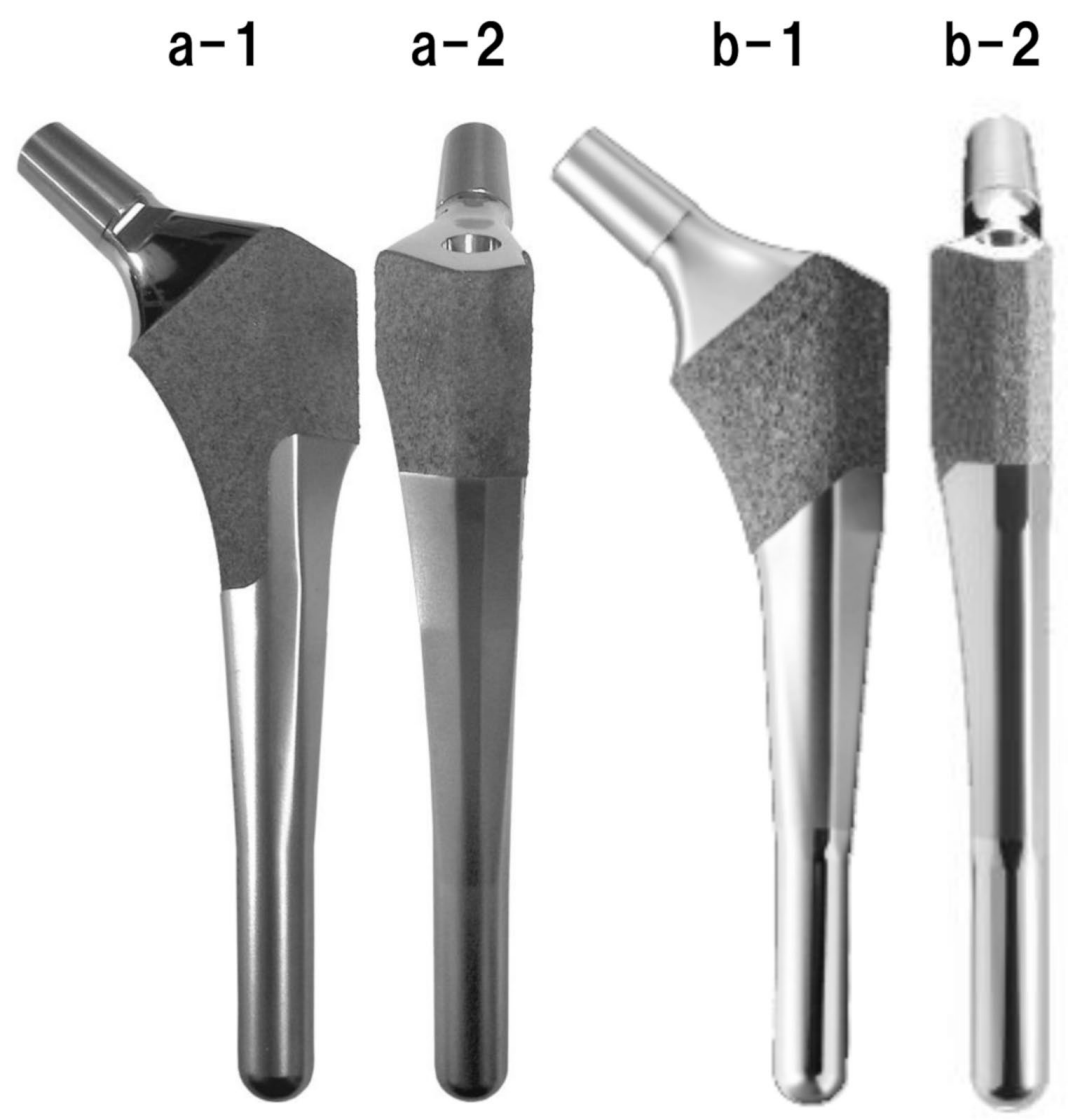
Shape of the stems. **(a)** FMS anatomic stem (KYOCERA Medical). (a-1) Frontal side of FMS anatomic stem. (a-2) Lateral side of FMS anatomic stem. **(b)** Anatomic Fit stem (KYOCERA Medical). (b-1) Frontal side of Anatomic Fit stem. (b-2) Lateral side of Anatomic Fit stem

The femoral stem design was based on the following requirements: (i) The distal portion must be straight, (ii) the distal stem diameter should not exceed the short-axis diameter of the medullary canal or the width of the isthmus, (iii) the stem length should not exceed the lower level of the femoral isthmus, (iv) the stem should have a proximal lateral flare and an optimal tapered shape to minimize stem subsidence and axial rotation, and (v) the medial portion of the stem should be wide enough to contact the medial femoral cortical bone to stimulate bone ingrowth into the hydroxyapatite coating (Fig. 2).

The FMS-anatomic stem had two proximal and six distal diameters each (medial radius: 68.0–104.0 mm; lateral radius: 95.0 mm; distal portion sizes: 9–12 mm by 1 mm). The Anatomic Fit stem was further improved in the third generation, with bone ingrowth occurring more proximally to reproduce physiological load transfer by decreasing the coated area to 35%, which is more proximal to the previous model. Additionally, the distal straight portion was polished to avoid distal fixation, and the lateral flare was reduced by 2 mm to lower the risk of fracture during insertion.

#### Implant survival and clinical assessment methods

The overall implant survival rate was assessed based on the need for stem revision. For clinical evaluation, the Japanese Orthopaedic Association (JOA) score was used preoperatively and at the final follow-up.

### Radiographic evaluation

Radiographic assessments included plain anteroposterior and lateral hip radiographs (Lauenstein) immediately post-surgery and at the latest follow-up. Preoperative medullary cavity shape and bone quality were evaluated using the Dorr classification [15]. Stem alignment was assessed in coronal and sagittal planes, with deviations exceeding 2° from the femoral axis considered malalignment [16–18].

Bone reactions, such as stem subsidence, radiolucent lines, osteolysis [19], pedestal signs [20], stress shielding [21], spot welds, and cortical hypertrophy [22], were investigated using plain radiographs at the final follow-up. Stem subsidence (in mm) was measured from the tip of the greater trochanter to the stem shoulder along the longitudinal axis [23]. Radiolucent lines exceeding 2 mm in thickness were noted, and their distribution classified according to Gruen zones [19]. The pedestal sign was defined as a shelf or endosteal new bone formation at the stem tip, partially or completely bridging the intramedullary canal [20]. Stress shielding was classified into four degrees, according to Engh et al., reflecting loading stress on the proximal femur [21]. Spot welds were defined as focal bone formations adjacent to the femoral stem, indicating bone ingrowth, and classified by Gruen zone [19]. Cortical hypertrophy was defined as the thickening of periprosthetic diaphyseal bone by ˃2 mm [23]. Radiographic loosening was identified if any of the following symptoms were present: (1) radiolucent lines ˃2 mm in thickness around the entire implant on the anteroposterior radiograph, (2) stem subsidence > 5 mm on serial radiographs, and (3) progressive varus or valgus tilt > 5° [21].

All radiographs were evaluated by a single observer using a Picture Archiving and Communication System.

### Data analyses

Statistical analyses were performed using GraphPad Prism 8 (La Jolla, CA, USA). Cumulative survival rates were calculated using the Kaplan–Meier method with 95% confidence intervals, with revision surgery for any reason as the endpoint.

Survival curves were compared between groups using the log-rank test. The chi-square test was used for radiographic results. A two-way ANOVA was used to compare Dorr classifications and stress shielding between stem types. Statistical significance was set at *P* < 0.05.

## Results

**Fig. 3:**
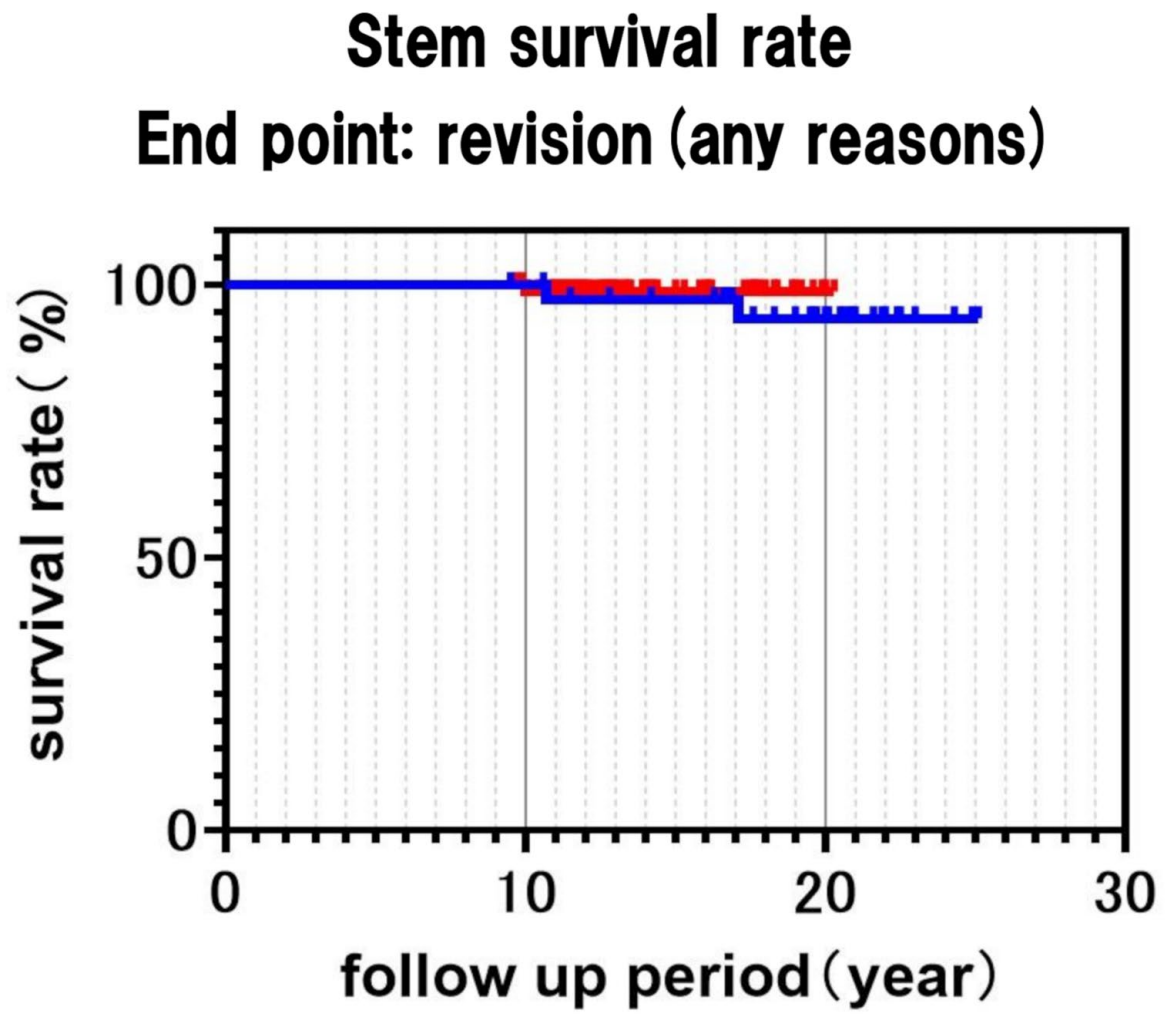
Kaplan-Meier analysis. Stem survival rate with end point of revision any reasons. Blue line is FMS anatomic stem and Red line Anatomic Fit stem (*p* > 0.05: Log rank test). Survival rate of 10 years of FMS anatomic stem and Anatomic Fit stem were both 100%. Survival rate of 15 years of both stems each were 97.2% and 98.8%. Survival rate of 20 years of both stems each were 94.4% and 98.8%

In the Kaplan–Meier analysis, survival rates of the FMS-anatomic stem at 15 and 20 years were 97.2 and 94.4%, respectively, with two revisions due to breakage at reduced neck at 17.1 and 10.8 years post-THA. The survival rate of the Anatomic Fit stem was 98.8% at both 15 and 20 years (Fig. 3). One patient required revision surgery due to habitual dislocation at 10 years. No significant differences were observed between the two stems.

Among the 34 hips with FMS-anatomic stems followed for over 10 years without revision (median, 20.1 years; maximum, 25.1 years), the mean JOA hip score was 82.5 (± 4.05) at the final follow-up (Table 2). Among 82 hips with Anatomic Fit stems with followed for over 10 years without revision (median, 13.1 years; maximum, 20.3 years), the score was 83.4 (± 5.05) at the final follow-up and there were no significant differences in JOA hip score between the two stems (*P* = 0.52).

**Table 2:** Comparison of clinical and radiographic data

Variables	Total	FMS-anatomic	Anatomic Fit	*p*-value (FMS-anatomic stem vs. Anatomic Fit stem)
Number of hips	119	36	83	
Revision for any reasons (%)	2.5(3/119)	5.6(2/36)	1.2(1/83)	0.17
Stem breakage (%)	1.7(2/119)	5.6(2/36)	0(0/83)	0.09
JOA score	83.1 ± 4.81	82.5 ± 4.05	83.4 ± 5.05	0.52
Coronal alignment (°)	0.51 ± 0.83	0.92 ± 1.03	0.34 ± 0.67	< 0.05
Sagittal alignment (°)	0.51 ± 0.88	0.36 ± 0.99	0.58 ± 0.82	0.88
Subsidence (mm)	0.30 ± 1.50	0.28 ± 0.91	0.31 ± 1.69	0.91
Radiolucent line(%)	1.6(2/119)	2.8(1/36)	1.2(1/83)	0.53
Pedestal sign(%)	28.5(34/119)	36.1(13/36)	25.3(21/83)	0.23
Stress shielding(%)				< 0.05 ^※2^
1	12.6(15/119)	11.1(4/36)	13.5(11/83)	
2	57.1(68/119)	72.2(26/36)	50.6(42/83)	
3	23.5(28/119)	13.8(5/36)	27.7(23/83)	
4	5.0(6/119)	2.7(1/36)	6.0(5/83)	
Spot welds (%)	99.1(118 /119)	100(36/36)	98.8(82/83)	0.51
Zone 1	34 (40/119)	17 (6/36)	41(34/83)	< 0.05
Zone 2	29 (34/119)	44 (16/36)	22 (18/83)	< 0.05
Zone 3	2 (2/119)	3 (1/36)	1 (1/83)	0.53
Zone 4	0 (0/119)	0 (0/36)	0 (0/83)	
Zone 5	0 (0/119)	0 (0/36)	0 (0/83)	
Zone 6	59 (70/119)	83 (30/36)	48 (40/83)	< 0.05
Zone 7	48 (57/119)	17 (6/36)	61 (51/83)	< 0.05
Cortical hypertrophy (%)	21.8(26 /119)	27.7(10/36 )	19.2(16/83)	0.30
Aseptic loosening	0 (0)	0 (0)	0 (0)	

Values are expressed as mean ± standard deviation or number (%)

※1 Formed site of spot welds, including zone 1.7 or none

※2 Above 3 degree of SSstress shielding

Fisher’s exact test or, Mann-Whitney U test was used

Coronal alignment averaged 0.92° (± 1.03) varus for FMS-anatomic stems, with 29 hips (80.5%) in a neutral position, compared to 0.34° (± 0.67) varus for Anatomic Fit stems, with 81 hips (97.6%) neutral (Table 2). Significant differences in coronal alignment were noted between the two stems (*P* < 0.05). Sagittal alignment averaged 0.36° (± 0.99) varus for FMS-anatomic stems, with 35 hips (97.2%) in a neutral position, compared to 0.58° (± 0.82) varus for Anatomic Fit stems, with 78 hips (94.0%) neutral.

For bone reactions, the mean stem subsidence in the FMS-anatomic stem was 0.28 mm (maximum, 4.6 mm), occurring within three months after primary THA with no further progression. In the Anatomic Fit stem, subsidence > 5 mm was found in one hip due to intraoperative fracture, but no progressive stem subsidence was observed after bone union at 15 mm. The mean stem subsidence for this stem was 0.31 mm (maximum, 15.0 mm) without progression and no significant differences in subsidence (*P* = 0.91). There were no cases of stem alignment change > 5° in the coronal plane from the initial implantation for either stem type. One hip showed a radiolucent line with a thickness of 1 mm in zone 3 of each stem. However, expansion occurred in none of the cases. Osteolysis was not observed in either stem type.

Pedestal signs were observed in 13 (33.3%), and 23 hips (26.7%) hips with the FMS-anatomic and Anatomic Fit stems, respectively without significant differences (*P* = 0.23).

**Fig. 4:**
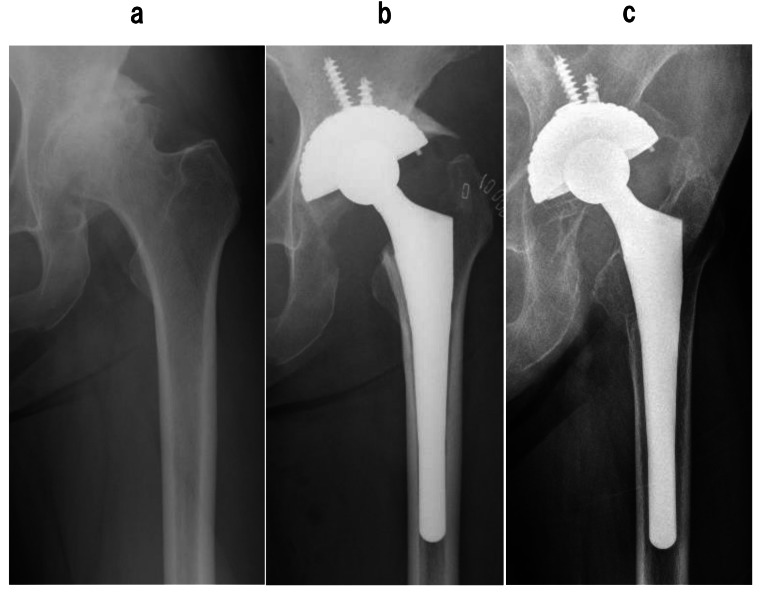
Stress shielding around anatomic fit stem. **(a)** Preoperative radiographs showed that femur were classified Dorr B. **(b)** Immediately after primary cementless THA, stem alignment was good position and canal filling was enough. **(c)** Followed up 21 years after primary operation, this femur was classified grade 3 stress shielding

Stress shielding, according to Engh’s classification, was observed as follows: for FMS-anatomic stems, grade 0: none, grade 1: four hips (11.1%), grade 2: 26 hips (72.2%), grade 3: five hips (13.8%), and grade 4: one hip (2.7%); and for Anatomic Fit stems, grade 0: none, grade 1: 11 hips (13.5%), grade 2: 42 hips (50.6%), grade 3: 23 hips (27.7%) (Fig. 4), and grade 4: five hips (6.0%). There were no significant differences in stress shielding between the two stem types (*P* < 0.05).

**Fig. 5:**
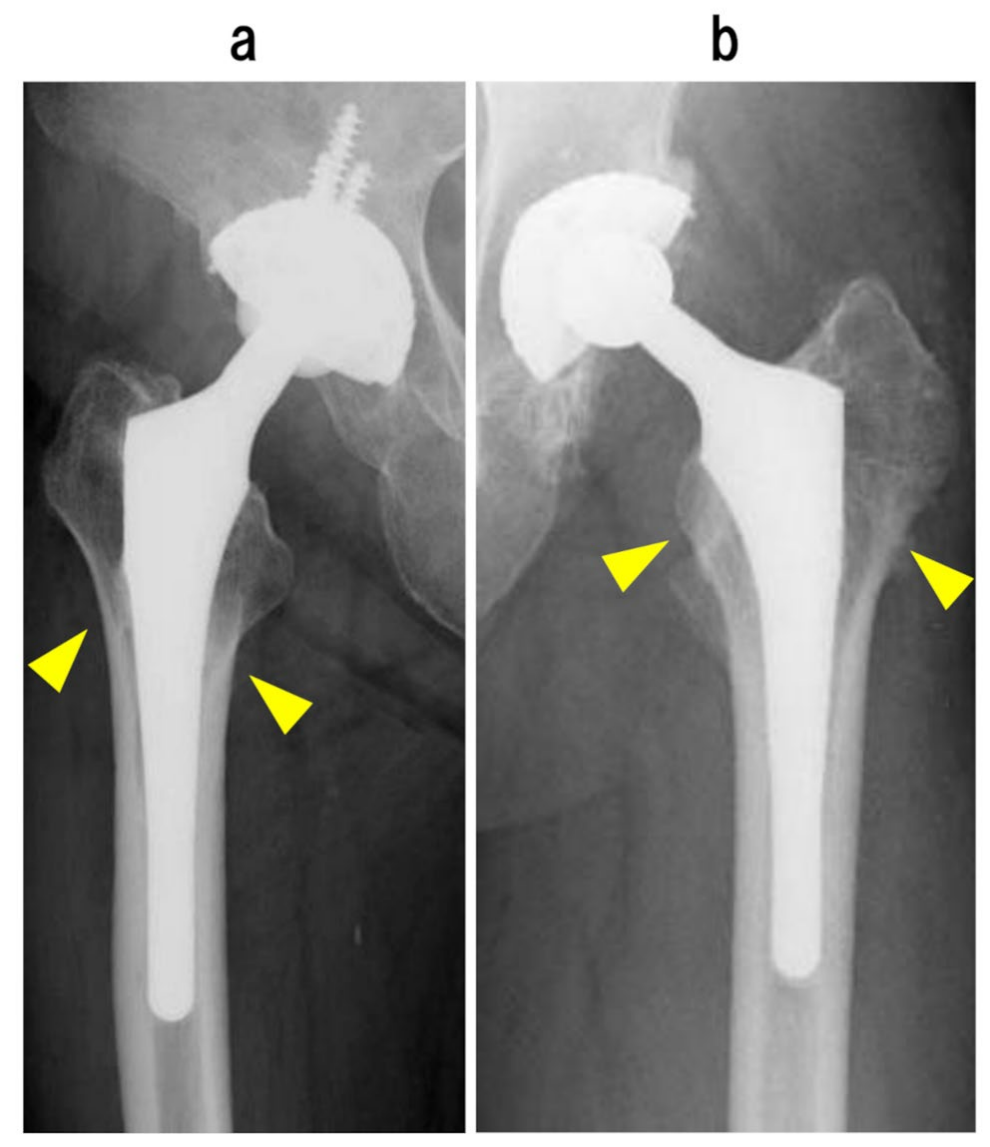
difference site of forming spot welds between fms anatomic stem and anatomic fit stem at anteroposterior radiographs. **(a)** Right side primary cementless THA with the FMS anatomic stem 12 years after and spot welds were formed at Gruen zone 2, 6. **(b)** Left side primary cementless THA with the Anatomic Fit stem 11 years after and spot welds were formed at Gruen zone 1, 7. The region of the spot welds was more proximal than that of the FMS -anatomic stem

Spot welds of FMS-anatomic stem group were observed in all 36 hips (100%) and mainly formed in zone 6 (83.3%). Spot welds of Anatomic Fit stem group observed in 82 hips (98.8%) and mainly formed in zone 7 (61.4%) in the Anatomic Fit stem. A significant difference was noted in spot weld positions between the two stem types (Fig. 5).

Cortical hypertrophy was observed in 10 hips (27.7%) with FMS-anatomic stems and 16 hips (19.2%) with Anatomic Fit stems, with no significant difference between the two types. No cases of aseptic loosening were observed for either stem category.

## Discussion

In this study, both types of stems, classified as Type 2 (fit and fill with a double wedge) and Type 6 (proximal anatomic shape) features according to Khanuja’s classification, showed a survival rate of over 95% at a minimum follow-up of 20 years. Both stems also showed favourable clinical outcomes. Radiographic evaluations revealed no clinical subsidence or malalignment, except in one case of intraoperative fracture. For stress shielding, grade 4 was observed in one hip with the FMS-anatomic stem and five hips with the Anatomic Fit stem. Both the stems exhibited good fixation and excellent long-term outcomes.

In this study, two of the 39 FMS-anatomic stems were revised for breakage, and one of the 86 Anatomic Fit stems was revised for recurrent dislocation. FMS-anatomic stems have a notch in the neck area, which seems to be structurally weak. The notch is trapezoidal in shape resulting in breakage with tensile stress [24]. The survival rate at 20 years for the FMS-anatomic stem and 15 years for the Anatomic Fit stem revision was 94.9 and 98.8%, respectively. The long-term results of Type 2 stems for 16 years postoperatively were 97.7–99.0% [25, 26], and those for Type 6 stems for 25–30 years postoperatively were 94.0–98.9% [27, 28]. Similarly, our results showed good survival rates. The high success rate of the Anatomic Fit stem can be attributed to two specific design features. First, the stem achieves initial stability through flare-fit fixation by making proximal contact. Second, the double-wedge and collarless implants with anterior and lateral flares provide additional rotational stability.

In this study, no cases of stem malposition were recorded in both groups of coronal alignment. However, there was a statistically significant difference (*p* < 0.005) in coronal alignment between the two groups. FMS anatomic stem group was implanted in varus position (0.92° ± 1.03) more than Anatomic Fit stem group (0.34°±0.67). We attribute this to the reduced proximal lateral flare of the Anatomic Fit stem, which allowed the alignment to be better defined in the distal canal. However, the difference in alignment did not affect clinical outcomes [29].

Previous reports using finite element analysis indicated that the shear stress of the Anatomic Fit stem was concentrated in the proximal area, especially on the border between the titanium arc spray and the smooth surface [12, 13]. In the current study, radiological data showed spot welds in almost all stems at the proximal quarter of the stem. While the FMS-anatomic and Anatomic Fit stems share similar geometry, except for the proximal arc-deposit surface, the Anatomic Fit stem exhibited more proximal spot welds than the FMS anatomic stem. This result has suggested that improvements in stem coating resulted in proximal spot welds as the bone grew, possibly leading to good long-term results.

For stress shielding, grade 4 was observed in the Anatomic Fit stem more than that in the FMS-anatomic stem, while the Anatomic Fit stem exhibited more proximal spot welds than the FMS-anatomic stem. Spot welds in the proximal region do not reduce stress shielding [30, 31]. Additionally, the Synergy stem (Smith & Nephew, Watford, UK), as a proximal filling stem, showed an 84% incidence of stress shielding [32]. Preoperative femoral bone quality is associated with severe stress shielding [33, 34]. In the present study, severe stress shielding over grade 3 occurred more frequently in the Anatomic Fit stem, which were used more frequently for Doll type B and C. Furthermore, Maeda et al. reported that the most proximal and medial calcar site-restricted fitting could help reduce stress shielding, emphasizing the importance of considering the ideal stem contact region to mitigate stress shielding [35]. However Anatomic Fit stem which was reduced lateral flare could result in more mismatch for distal to proximal femoral canal than FMS-anatmoic stem .

This study has some limitations. First, the sample size was relatively small, although there was sufficient statistical power to confirm similar migration between the two groups. Additionally, a pure comparison could not be made because the follow-up periods for Anatomic Fit and FMS Anatomical were quite different. Therefore, our findings may not apply to other stem models.

## Conclusion

The survival rates of the FMS-anatomic and Anatomic Fit stems were excellent during the long-term follow-up. While improvements in stem design allowed for controlled spot-weld positioning and enhanced bone ingrowth, the more proximal coating design did not significantly reduce stress shielding. Further long-term follow-up is needed to determine the clinical impact of the differences between the two stems.

## Data Availability

No datasets were generated or analysed during the current study.
